# On the Disposition of Time and Its Relation to Fees

**Published:** 1884-04

**Authors:** A. W. Harlan

**Affiliations:** Chicago, Ill.


### ON THE DISPOSITION OF TIME AND ITS RELATION TO FEES

BY A. W. HARLAN, D. D. S., CHICAGO, ILL.

The disposition of time by allotment to patients is, especially to a young dentist, very easy of accomplishment; but a time arrives in his history, if he has attained the practice which always comes to the earnest student and industrious worker, when it sadly perplexes him, and unless he is unusually methodical, he is apt to undertake a great many operations without much thought that one appointment may be so limited that he will be free for undertaking the next. This is a question which has given me no little concern, and the only reason I have chosen it for my theme has been to get a free and full interchange of views from gentlemen, many of whom are my seniors in age and experience, that in future I may be enabled to extricate myself from some of the dilemmas in which want of time may involve any of us.

Operating, as I have done for years, a certain number of hours daily at the chair, I am forced to a personal study of this subject.

I have not gained much information from my brethren as to how they conduct a practice; so I have adopted my present method from necessity. My day usually begins at eight-thirty A. M., and I have found from long experience that as a rule it is best to have a male patient to commence with, and an appointment of one hour to one hour and a half is generally made. It is devoted to the preparation of cavities, cleaning teeth, or the removal of a pulp, or filling a root, and the insertion of the simpler kinds of fillings. It may be for the fitting of an artificial crown, or its adjustment. I do not at this early hour begin a large contour filling; first, because it frequently happens that persons who have broken a tooth, or who have the toothache, are liable to come in early, and they need a little temporary attention. Secondly, if they do, you can arrest your early labors by having a chair in an adjacent room, and give the necessary moments which might not be possible were you intensely occupied. Another reason for engaging the first hour or so in the manner indicated, is that in cities it is frequently cloudy or dark so early in the morning, and of course that is a bar to the accomplishment of really fine operations. At nine-thirty A. M., or ten o'clock, the heavy work of the day begins; then the large fillings are started, and the difficult cavities are filled; or it may happen that I wish to fill three or four proximal cavities; operations that require extra good light. This usually requires one and a half to two and a half hours, the average being two hours. The next heavy work is then begun, which consumes the time up to one, or one-thirty P. M.; then lunch; after which perhaps another large filling, or very likely two; if the latter, the time up to four is consumed; if only one filling is made, I then have another appointment at three, for a simple filling or two, or three or four amalgam or tin fillings are inserted. Then I begin to change wedges, to attend to my regulating cases, to treat abscesses, cases of pyorrhœa, finish fillings previously made, and attend to the numerous miscellaneous cases that every dentist of full practice has to see, examine teeth, make temporary fillings, extract a root or tooth, give advice, or write a prescription. Then I charge the labors of the day and make credits for cash received, make out bills and write letters, etc.

This ends the day—in winter at five to five-thirty P. M., and in summer at five-thirty to six P. M. The time is too long, but how to shorten it and meet the demands of an increasing practice, try experiments, study and keep *au courant* with all that is going on in the dental world, take a peep at current medical literature, attend societies, read papers that one has written, deliver a course of lectures or two, assist in organizing new societies or colleges, take needed recreation, attend to social and home duties, and still earn enough to run the financial wheels and save something besides, is the question. Seriously, to view this matter in any light is discouraging. The only way in which and by which any practitioner with a conscience can long be successful and do justice to his patrons, is to raise his fees. What shall be the basis for fees? I take it that the demand for services in a legitimate practice, by the public, is made by it to the professional man on account of his supposed skill, honesty, and ability to treat successfully the ills which negligence, accident, misfortune or ignorance has made it to suffer.

If the dentist satisfies the public, his patrons, that he does his level best in every instance, they trust him, they flock around him, and overwhelm him with their patronage. The greater number of those who seek the services of a dentist are not the possessors of large incomes. If it were so, in a few years a dentist might retire with a competence. How many have retired in Chicago? People with growing families and the possessors of moderate means are the most constant in their visits to the dentist. Families of large wealth are found in many places of recent origin. Many of them are, from their previous surroundings or education, unable to discriminate between the skilled and the unskilled. They are restive and foolish in their unwillingness to bear with the necessary infliction of pain to treat or fill teeth properly. They are often unwise enough to offer to compensate for services of the most wearing and tedious nature in the spirit of dispensers of charity, or consider that they have done what was perfectly right in offering to pay half the bill for a receipt in full, and you are given the inestimable privilege at that cost of retaining them as your clients, or you are invited to collect through the courts, which is usually a poor way of obtaining one's dues on account of the comparative smallness of the claim.

I think that in disposing of time the most valuable should be allotted to those who appreciate one's labors, and who are willing to compensate for them. If one undertakes a difficult and delicate operation for people not able to compensate in the fullest manner, we are bound to do for those persons the same as though the full compensation had been received.

My plan of allotting time is to parcel it out to patients as far as may be without too great inconvenience, so that the best hours of the day are devoted to the cases needing most care and skill, having some reference to the appreciation of the patient and his willingness to make proper compensation for the service rendered. If I were to estimate the value of time at so many dollars per hour, I should say that an hour from eight-thirty to nine-thirty is worth (according to the demand for time of the operator) about four to six dollars; from nine-thirty to one-thirty about five to eight dollars; from two to four P. M. about five to seven dollars; and four to five-thirty at three to six dollars per hour. I do not believe in the idea of receiving compensation by the hour, as it is unfair to patient and operator. Regular routine operations generally take up two-thirds of the time of every dentist. If he operates exclusively, he sees more patients daily than the one who spends one-fourth to one-half his time in the prosthetic department; the former, from habit, or application, or increasing dexterity, can make a larger number of operations daily than his brother who is not so constantly occupied. He learns from experience to manage his patients better, so as to expedite matters. He makes no false motions, everything is in readiness to work with, all his materials are at hand, his gold, or tin, or Robinson filling, his gutta-perchas and plastics are all ready to be used. While the operator without system or method is getting his patient seated and his implements ready, the methodical operator has adjusted the dam, prepared a cavity and filled it, received his fee, and is ready for the next patient. System, order, and celerity of motion tell in a long day.

If, on account of your method of managing a patient and the wisdom you acquire in repeatedly doing the same kind of operations, you are able to insert two fillings where your slower brother inserts but one, it is an injustice to you to be paid the same fee for the performance of double duty. I believe, also, that a habit of making rapid operations tends to a greater thoroughness in the vast majority of cases. There is no uncertainty about the how or where of cutting. The operator who wishes to accomplish much has sharp chisels and good excavators, and his burrs are not dull. He spends little time in changing engine bits, because the cavity is well opened before he uses a burr; it is saturated with the obtunder, no matter what, quickly dried, a spoon-shaped instrument is dexterously applied, and presto, the cavity is ready for filling. When a cavity difficult of access and more difficult to fill is met with, even though it be small, if it consumes his vitality rapidly, the operator should make his fee commensurate with the skill required for its successful accomplishment, rather than on the basis of size of cavity or time required for its filling. Skill and knowledge and judgment are the factors to be considered in formulating a tariff of fees, and not time, nor materials, nor a desire to charge so much per diem.

In concluding this paper I desire to say that a patient has some rights which every dentist ought to respect. I do not permit any interruption by callers for appointments, or chance visitors; the only case which comes to me from nine-thirty to four which I stop to see (as a rule), is one of actual suffering. I make it known to patients on my appointment card that they can come at four and take their turn any day, and usually they acquiesce in this arrangement. A practitioner in a large city may lose a few patients at first, but those who make appointments appreciate the fact that they are required to spend only about one-half or two-thirds the time which they might have spent had the dentist been talking to half a dozen persons during the preparation and filling of a cavity, requiring at the utmost one hour for its completion. Time, to a dentist who has labored to perfect himself in all the details of his practice, is valuable; and he stands in his own light when he thoughtlessly or carelessly consumes it, to his own detriment and against his patients' interests, by endeavoring to make it up, as many are in the habit of doing, by improperly preparing cavities, impacting gold, finishing fillings poorly, or half doing many other important operations not necessary to enumerate, solely on account of a lack of system, method, or business-like habit of disposing of time.

